# Potential-Resolved Electrochemiluminescence and Its Application in Disease Biomarker Detection

**DOI:** 10.3390/bios15110749

**Published:** 2025-11-07

**Authors:** Liangbiao Wang, Xiaojing Su, Rongrong Han, Dexin Du, Mingquan Guo

**Affiliations:** 1 School of Medicine, Anhui University of Science and Technology, Huainan 232001, Anhui, China; yunshan@mail.ustc.edu.cn; 2 Key Laboratory of Industrial Dust Deep Reduction and Occupational Health and Safety of Anhui Higher Education Institutes, Huainan 232001, Anhui, China; 3 Anhui Province Engineering Laboratory of Occupational Health and Safety, Huainan 232001, Anhui, China; 4 Department of Neurology, Centre for Leading Medicine and Advanced Technologies of IHM, The First Affiliated Hospital of USTC, Division of Life Sciences and Medicine, University of Science and Technology of China, Hefei 230001, Anhui, China; sxjing20@mail.ustc.edu.cn; 5 School of Mechanics and Photoelectric Physics, Anhui University of Science and Technology, Huainan 232001, Anhui, China; 2024179@aust.edu.cn; 6 Department of Chemistry, University of Science and Technology of China, Hefei 230026, Anhui, China; dxdu@mail.ustc.edu.cn

**Keywords:** potential-resolved, electrochemiluminescence, biosensors

## Abstract

Electrochemiluminescence (ECL) is a chemiluminescence phenomenon triggered by electrochemical reactions at the electrode surface, which has gradually become a high-sensitivity detection technology due to its low background, simple instrumentation, and high sensitivity. Therein, potential-resolved ECL refers to the generation of two or more ECL signals with distinct potentials and wavelengths during an electrochemical process. This unique capability enables simultaneous multi-signal outputs, making potential-resolved ECL particularly promising for self-calibration and multiplexed detection strategies. In this review, we focus on two critical aspects: on the one hand, the advancement of traditional ECL luminophores and potential-resolved ECL systems was reviewed, which were classified, respectively, into three categories to be introduced in detail (inorganic, organic and nanomaterial-based ECL luminophores or potential-resolved ECL of metal–organic complexes, layer-by-layer-modified electrodes, and nanomaterials). On the other hand, we summarized ECL detection strategies based on potential-resolved ECL systems and the application of these protocols in disease biomarker detection, which results in two categories (self-calibration strategies and multi-target strategies) for discussion. In this work, we aim to inspire investigators to explore novel ECL luminophores and design detection strategies with high performance, which could provide strong support for precision medicine, personalized assessment, portable medical devices, and the digital transformation of healthcare.

## 1. Introduction

Chemiluminescence (CL) is the emission of light resulting from a chemical reaction, typically an oxidation process, which produces an electronically excited intermediate or product that subsequently relaxes to its ground state by emitting photons [1]. This phenomenon provides a high signal-to-noise ratio for sensing applications as it does not require an external light source for excitation, thus avoiding issues of background light scattering and autofluorescence. CL-based assays have been widely exploited in various fields, particularly in clinical diagnostics and immunoassays [2,3]. Electrochemiluminescence (ECL) refers to chemiluminescence generated at electrode surfaces, which is controlled by electrochemical reactions [4,5]. In a typical process, electrolysis at the electrode produces reactive intermediates, which undergo subsequent electron transfer to generate excited states; subsequently, light emission occurs as these species relax back to the ground state [6]. Dufford and coworkers first reported the ECL phenomenon in 1927 [7], and it has gradually become a high-sensitivity detection technology due to its low background. Over the past century, ECL has evolved into a powerful analytical technique with broad applications in medicine, environmental monitoring, biology, and analytical chemistry [8,9,10,11,12,13,14,15].

ECL synergistically combines the advantages of its parent techniques. From electrochemistry, it inherits exquisite temporal and spatial control over the light-emitting reaction simply by switching the electrode potential. From chemiluminescence, it retains the core benefit of high sensitivity due to the absence of an external light source, which eliminates scattering and autofluorescence background. By uniting the merits of electrochemistry and chemiluminescence, ECL offers several unique advantages: (1) With the same conventional chemiluminescence, ECL does not demand an external excitation light source, yielding intrinsically low background and high sensitivity. (2) The ECL excitation is generated by electrical energy, which expands stable chemicals as alternative co-reactants instead of activated species, thereby enhancing stability and reproducibility. (3) Electrochemical processes can convert diverse reaction species into ECL-active intermediates, broadening the range of detectable targets. (4) The electrochemical process is controlled by potential, which allows precise regulation of the spatial and temporal characteristics of emission, enabling highly selective, sensitive, and high-throughput assays. (5) The synergy between electrochemical control and optical detection is particularly powerful for investigating reaction mechanisms. For instance, by correlating the applied potential with the onset of light emission (e.g., using a CCD grating spectrometer to acquire ECL spectra synchronized with electrochemical signals), researchers can pinpoint the exact potential window where key intermediates (e.g., radical ions) are generated and react, providing direct evidence for reaction pathways that are difficult to obtain by either method alone. Compared to other analytical approaches, ECL offers unique advantages, including simple instrumentation, minimal background interference, and exceptional sensitivity, making it a fundamental tool for the ultrasensitive detection of biomarkers [16,17].

ECL luminophores are highly diverse. To systematically introduce them, they are commonly and broadly classified into several categories, with inorganic, organic, and nanomaterial-based luminophores. Inorganic ECL luminophores mainly refer to ‘metal–ligand’ complexes formed by transition metals such as ruthenium, iridium, and osmium coordinated with organic ligands. Organic ECL luminophores primarily consist of organic compounds containing localized conjugated structures, such as hydrazides, acridinium derivatives, polycyclic aromatic hydrocarbons, and oxalate esters. Emerging nanomaterial-based ECL luminophores, including nanoclusters, quantum dots, and metal–organic frameworks, among others, can yield ECL with high quantum efficiency. In recent years, benefiting from the unique chemical, physical, and physicochemical properties of nanomaterials, the behaviors of ECL nano-luminophores have been extensively investigated, and their applications have rapidly advanced [18,19,20]. Currently, the modification and engineering of existing ECL luminophores represent a major research focus in this field. Reported strategies mainly fall into the following categories: (1) nanomaterials integrated with ECL-active molecules possess excellent stability and biocompatibility to generate high-performance luminophores, such as Ru-doped SiO_2_ nanoparticles and *N*-(4-aminobutyl)-*N*-ethylisoluminol (ABEI)-functionalized Au nanoparticles [21,22]. (2) The nanomaterials are designed and modified with specific functional groups on the surface, for example, by designing core–shell-structured nanomaterials or introducing -COOH/ions onto their surfaces. (3) The different luminophores are assembled into combinations through covalent bonding, hydrogen bonding, or other intermolecular interactions to achieve potential-resolved, wavelength-resolved, or color-resolved ECL systems. This research has significantly expanded the repertoire of ECL luminophores and established an important foundation for their analytical applications. Despite the remarkable progress, most reported ECL luminophores are still limited to single-emission systems. The development of novel nanomaterial-based luminophores with multiple ECL emissions is crucial for advancing detection technologies and expanding the scope of analytical applications.

Potential-resolved ECL indicates the generation of multiple potential-resolved ECL signals by a single electrochemical process in an ECL system. This field was launched in 2004 with the discovery by Brian D. Muegge and co-workers of multicolor ECL phenomena in mixed systems of Ru^2+^ and Ir^3+^ complexes in organic media. However, its practical use was limited by the incompatibility of organic solvents. Subsequent studies improved potential-resolved ECL systems by immobilizing different luminophores onto electrode surfaces through layer-by-layer assembly, which enabled ratiometric detection and multiplexed analyte monitoring. Nevertheless, these strategies suffered from drawbacks such as label dependence and complex and time-consuming electrode preparation. In recent years, nanomaterials with potential-resolved ECL have attracted increasing attention as a simpler and more versatile alternative. These materials can produce multiple ECL emissions in a single electrochemical event, offering significant potential for self-calibration and simultaneous multi-analyte detection [23]. For self-calibration, ratiometric or differential signal processing can effectively eliminate system errors introduced by environmental factors such as matrix effects, scattering, or temperature fluctuations. For multiplexed detection, distinct ECL emissions can be assigned to different analytes, thereby improving efficiency and reducing cost. Since the first report of potential-resolved ECL nanomaterials by Mahdi Hesari in 2015, researchers have explored additional studies on potential-resolved ECL [24,25,26]. Moreover, applications of these nanomaterials in self-calibrating assays and multiplexed detection remain scarce. Therefore, the continued development of potential-resolved ECL nanomaterials and the exploration of their applications are of great importance for advancing analytical detection technologies.

**Scheme 1:**
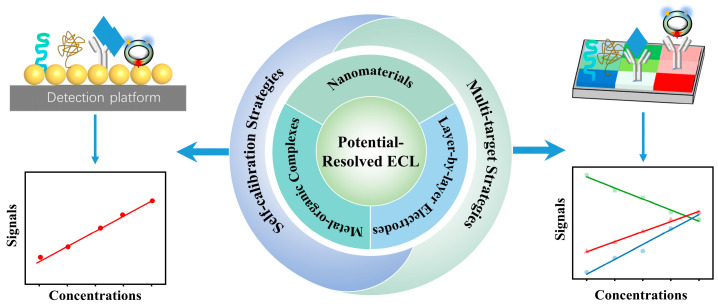
Potential-resolved ECL luminophores and its applications.

Depending on potential-resolved ECL luminophores that have been discovered and exploited, this work discussed traditional ECL luminophores (inorganic ECL luminophores, organic ECL luminophores and nanomaterial-based ECL luminophores) and potential-resolved ECL luminophores (potential-resolved ECL of metal–organic complexes, layer-by-layer-modified electrodes, and nanomaterials), divided into three categories (Scheme 1). And then, we compared these ECL luminophores between traditional ECL luminophores and potential-resolved ECL luminophores. The obvious advantages were indicated for multiple ECL signals in a potential-resolved ECL system. Based on this, a strategy for self-calibration and simultaneous multi-target detection can be developed for biomarkers. We have summarized these ECL luminophores and detection strategies for biomarkers, which we expect to inspire investigators to explore novel ECL luminophores and design detection strategies with high performance.

## 2. ECL Luminophores and Potential-Resolved ECL Luminophores

In recent years, potential-resolved ECL has emerged as a research hotspot. Compared to traditional ECL luminophores, this system can produce multiple emissions at distinct potentials, offering significant application potential that can be applied generally in areas such as multiplexed detection, self-calibrated assays, and the development of multicolor light sources. To date, three main types of potential-resolved ECL systems, based on the modification and integration of conventional ECL luminophores, have been reported. The first type is metal–organic complex-based systems, including mixtures of Ru(II) and Ir(III) complexes as well as Ru(II)-/Ir(III)-bridged coordination compounds. The second type is layer-by-layer electrode assembly, in which different ECL luminophores are sequentially immobilized onto electrode surfaces, thereby enabling potential-resolved multicolor ECL emission. The third type is nanomaterial-based potential-resolved ECL, in which luminescent molecules, nanoscale luminophores, or luminophores are combined through co-functionalization, doping, or enrichment strategies to generate multiple emissions. In this section, we first discussed traditional ECL luminophores and then summarized the research progress for potential-resolved ECL.

### 2.1. Traditional ECL Luminophores

ECL luminophores are diverse, encompassing traditional luminescent materials such as luminol and its derivatives, luminol analogs, oxalate esters, and bipyridyl compounds, as well as nanomaterial-based luminophores including nanoclusters, quantum dots, and metal–organic framework (MOF) luminophores. This article classifies commonly used ECL luminophores into three categories: inorganic luminophores, organic luminophores, and nanomaterial-based luminophores.

#### 2.1.1. Inorganic ECL Luminophores

**Figure 1:**
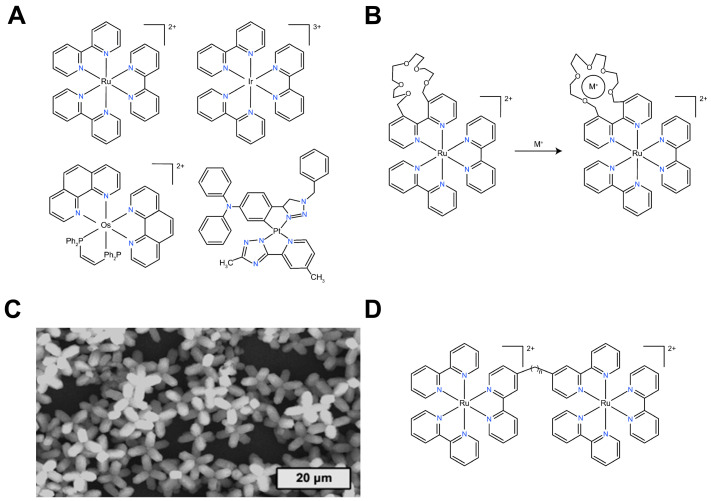
(**A**) Common metal–organic complex-based ECL luminophores; (**B**) modification of specific functional groups on the ligands of Ru(bpy)_3_^2+^; (**C**) SEM of supramolecular assemblies of Ru(bpy)_3_^2+^ [36]; (**D**) ECL luminophore based on multi-center Ru(II) organic complexes.

Inorganic ECL luminophores primarily refer to a class of ‘metal–ligand’ complexes formed by transition metals such as ruthenium, iridium, and osmium, coordinated with organic ligands (Figure 1A). Among these, Ru(bpy)_3_^2+^ and its derivatives have become a research hotspot due to their excellent stability, outstanding ECL performance, and high ECL efficiency in aqueous solutions. Ru(bpy)_3_^2+^ can react with compounds containing groups such as -NH_2_, C_2_O_4_^2−^, and S_2_O_8_^2−^ to generate ECL, which has been applied in the detection of various substances, including alkyl amines, oxalate esters, ascorbic acid, amino acids, disease biomarkers, nucleic acids, and pharmaceuticals [27,28,29,30,31,32,33,34,35]. Ru(bpy)_3_^2+^ is the most widely studied inorganic ECL luminophore, which can generate ECL through both annihilation and co-reactant pathways. The annihilation-type ECL of Ru(bpy)_3_^2+^ is used to study the principles of electrochemical reactions, while the co-reactant-type ECL in aqueous solutions has been extensively applied in practical analysis and detection. Among these, the Ru(bpy)_3_^2+^/TPrA system is widely used due to its exceptionally high ECL efficiency, providing extremely sensitive ECL labels at the pmol/mL level. This system enables highly sensitive and accurate detection of targets over a concentration range exceeding six orders of magnitude. Consequently, the Ru(bpy)_3_^2+^/TPrA co-reactant system has found broad applications in commercial ECL-based immunoassays and DNA analysis.

Although the Ru(bpy)_3_^2+^/TPrA system is widely used, it still has several limitations that restrict its ability to meet the practical demands of research and detection. These limitations include a small molecular size, which makes it difficult to purify and separate, the lack of suitable functional groups for target labeling, and the difficulty in functionalizing the other ECL luminophore. As a result, several improvements are typically made to Ru(bpy)_3_^2+^ in practical analytical studies: (1) modifications are made for ligands of Ru(bpy)_3_^2+^, such as the incorporation of amine or carboxyl groups for easier binding or partial ligand replacement (Figure 1B) [37]; (2) Ru(bpy)_3_^2+^ is assembled into nanomaterials such as supramolecular structures (Figure 1C) [36]; (3) Ru(bpy)_3_^2 +^ is doped into nanomaterials; and (4) other metal–organic compounds are combined to form multi-metal Ru(II) complexes (Figure 1D) [38]. In terms of ligand design or modification, various attempts have been made to endow the molecule with the capability to recognize metal ions. For example, crown ether or phenanthroline groups covalently bonded to the bipyridine ligand are used for sensing metal cations (Figure 1B) [37,39,40], which can help study the effects of metal cations on the ECL annihilation or co-reactant pathways of Ru(II). Additionally, substituting some of the ligands in Ru(bpy)_3_^2+^ can impart unique properties. For instance, replacing one of the bipyridine ligands in Ru(bpy)_3_^2+^ with 2,2′-bipyridine and [3,2-a:2′,3′-c] phenazine (dppz) results in the [Ru(bpy)_2_dppz]^2+^ complex, which exhibits a high affinity for DNA [41], making it suitable for DNA analysis and detection. Subsequently, self-assembly of Ru(bpy)_3_^2+^ into supramolecular structures was achieved by mixing H_2_PtCl_6_ and Ru(bpy)_3_Cl_2_ aqueous solutions, which led to the formation of supramolecular assemblies (Figure 1C) [36]. These structures displayed excellent ECL behavior, making them promising candidates for novel ECL materials. In recent years, the combination of Ru(bpy)_3_^2+^ or functionalized Ru(bpy)_3_^2+^ with nanomaterials has yielded numerous high-performance ECL luminophores, which have been widely applied in the analysis and detection of real samples [42,43,44]. To enhance the ECL sensitivity of the system, researchers have designed and synthesized multi-center Ru(II) complexes (Figure 1D), using bridging ligands to connect multiple Ru(II) complexes, generating stronger ECL signals [38,45,46,47,48]. The first reported multi-center Ru(II) complex was a bi-center complex, which exhibited a 2–3 fold increase in ECL intensity. Following this, a series of multi-center Ru(II) complexes were synthesized and studied, further enhancing the ECL efficiency of the ‘Ru(II)–organic complex’ system.

Despite the high ECL activity of many neutral Ir(III) complexes in non-aqueous environments, often exhibiting much higher luminescent efficiency than Ru(bpy)_3_^2+^, their poor solubility in aqueous solutions and sensitivity to oxygen have limited their practical applications. Recent reports have demonstrated that insoluble neutral Ir(III) complexes encapsulated in SiO_2_-PEG nanomaterials result in stable ECL behavior in aqueous solutions [49]. This protocol enhances the solubility of Ir(III) in water. It reduces oxygen quenching, providing a promising route for obtaining water-soluble ECL materials that open new avenues for ECL-based biological sample analysis. Recent studies on the ECL properties of Ir(III) complexes have found that some of these Ir(III) complexes exhibit good solubility in aqueous solutions and display ECL intensities comparable to that of Ru(bpy)_3_^2+^ [50]. This research has expanded the application potential of Ir(III) complexes in ECL detection. Furthermore, studies have shown that other heavy metal–organic complexes, such as osmium and platinum compounds, also exhibit ECL properties. However, these luminophores can only produce ECL in organic solvents, and their relatively low luminescent efficiency and high cost have hindered further research and practical applications.

#### 2.1.2. Organic ECL Luminophores

**Figure 2:**
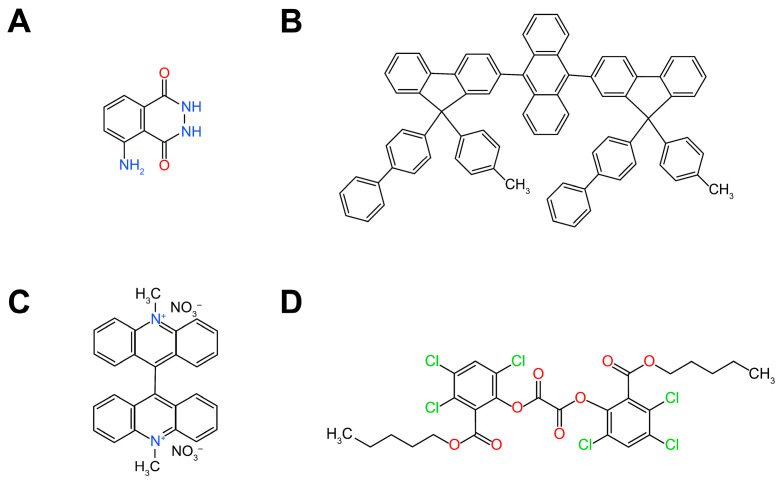
Different types of organic ECL luminophores: (**A**) hydrazide compounds: luminol; (**B**) polycyclic aromatic hydrocarbons; (**C**) acridinium derivatives: lucigenin; (**D**) oxalate esters: bis (oxalate) ester.

Organic ECL luminophores primarily include hydrazide compounds, acridinium derivatives, polycyclic aromatic hydrocarbons, and oxalate esters. Below, we introduce each of these luminophores: Hydrazide compounds refer to organic compounds with the general molecular formula ‘RCONHNHR_1_’. Among hydrazides, luminol and its derivatives are the most widely used ECL luminophores in practical applications and research (Figure 2A). The ECL phenomenon of luminol was first discovered by Albrecht et al. in 1928 [51]. Numerous subsequent studies have investigated the ECL properties of luminol and its derivatives [52,53,54,55,56,57]. Extensive research has been conducted on the ECL mechanism of luminol. In basic media, luminol loses a proton and is electrochemically oxidized at the electrode surface to form an anion. The resulting diazo compound undergoes further oxidation in the presence of hydrogen peroxide, generating the excited-state 3-amino-phthalate ester, which returns to the ground state to emit ECL. In facilitating the basic conditions of proton loss, the pH of the aqueous medium plays a significant role in the ECL of luminol. Studies have shown that the ECL efficiency of luminol increases with pH, typically reaching its maximum at around pH = 11. In practical applications, different types of catalysts, such as enzymes (horseradish peroxidase) and metal ions (Co^2+^, Fe^3+^), are commonly used to enhance ECL efficiency. Research indicates that Co^2+^ exhibits the best catalytic effect in the luminol system.

According to IUPAC, polycyclic aromatic hydrocarbons (PAHs) are aromatic compounds characterized by a fused polycyclic (or fused-ring) system. This means their structure consists of multiple rings that share pairs of atoms, resulting in a highly conjugated and stable planar molecular framework. Polycyclic aromatic hydrocarbons are among the earliest studied ECL luminophores and are considered a classic ECL system (Figure 2B) [53,58,59,60,61]. The ECL mechanism of polycyclic aromatic hydrocarbons can be described as follows: Initially, polycyclic aromatic hydrocarbons (***R***_1_, ***R***_2_) are oxidized or reduced at the electrode surface to form radical ions (${R_{1}}^{•-}$, ${R_{2}}^{•-}$). In a sufficiently energetic reaction system, these radical ions undergo singlet-state mechanisms.

(1) $$R_{1}+{\;e}^{-}={{\;R}_{1}}^{•-}\;$$

(2) $$R_{2\;}-{\;e}^{-}={{\;R}_{2}}^{•+}$$

(3) R1•−+R2•+=R*11+R2

The radical ions ${R_{1}}^{•-}$ and ${R_{2}}^{•-}$ then undergo an annihilation reaction, resulting in the formation of an excited-state species, R*11. Here, ***R***_1_ and ***R***_2_ refer to either the same compound or two different precursors of the same compound. In energy-deficient reaction systems, triplet-state reactions may occur.

(4) R1•−+R2•+=R*13+R2

(5) R*13+R*13= R*11+R1

In these two pathways, the excited species can be either R*11 or R*13, depending on their relative energies. In energy-rich systems, both pathways can occur, but the singlet-state pathway typically dominates. Polycyclic aromatic hydrocarbons (PAHs) are environmental pollutants found in air, soil, and water. PAHs originate not only from their intentional synthesis on a large scale for high-value applications but also, primarily as environmental pollutants, from the incomplete combustion of organic materials such as industrial waste and fossil fuels [62,63]. Some of these compounds exhibit severe mutagenic and carcinogenic properties and are classified as priority pollutants by both the European Union and the United States [64,65]. Therefore, the use of ECL technology for the highly sensitive detection of PAHs plays a crucial role in environmental protection.

Acridinium derivatives are planar compounds with a π-conjugated system and nitrogen-containing heterocycles. Acridine and its derivatives (Figure 2C) are widely used in pharmaceuticals and industry, playing an important role in spectral analysis. Lucigenin (1935) was the first reported acridinium compound with ECL and has been extensively studied and applied [66,67,68]. Since then, acridinium compounds have attracted significant interest in the design of new ECL luminophores due to their high ECL efficiency in aqueous solutions and ease of synthesis, which promotes research into the ECL properties and mechanisms of acridinium compounds. Under alkaline conditions, hydrogen peroxide oxidizes acridinium compounds to generate an intermediate, dioxacyclobutanone, which further decomposes into the excited-state *N*-methylacridone, which then returns to the ground state and is accompanied by light emission. As a highly efficient ECL luminophore, luminol has been widely applied in various fields, such as metal ion and organic compound detection (e.g., superoxide radicals in neutral or weakly alkaline solutions, pH = 7–10), and biological tissue sample analysis (e.g., hydrogen peroxide and enzyme systems). Compared to Ru(bpy)_3_^2+^ and luminol, acridinium compounds and their derivatives have some drawbacks, including higher cost, irreversible electrochemical reactions, and fewer co-reactants, which make them less popular than the former two. Notably, acridinium compounds can be functionalized with various groups to modulate their properties, including color, luminescence efficiency, and molecular recognition ability, offering substantial potential for future development. The tunable color property makes acridinium compounds highly promising for multiplexed analysis of multiple targets, and the development of new co-reactants and electrocatalysts (such as nanomaterials) will further expand their application scope.

The peroxydioxalate luminescence system (Figure 2D) is currently the second most efficient luminescent system after bioluminescence, with the highest quantum yield reaching 34% [69]. The ECL reaction between various oxalates and oxalamides with hydrogen peroxide is referred to as the chemiluminescence of the peroxydioxalate system. The first peroxydioxalate luminescent system, involving the reaction of oxalyl chloride with hydrogen peroxide in the presence of a fluorophore such as 9,10-diphenylanthracene, was discovered by Chandross in 1963 [70]. The luminescent reaction of peroxydioxalates proceeds with electron transfer from the fluorophore to the oxalate dianion intermediate, which then decomposes into CO_2_ and ${{\mathrm{CO}}_{2}}^{•-}$. The carbon dioxide radical anion subsequently transfers an electron to the high-energy fluorophore, generating light emission [69,71]. While the peroxydioxalate system exhibits excellent chemiluminescent properties and has been widely applied in the analysis of biological samples, its ECL performance is not as prominent. As a result, there has been limited interest in studying its ECL properties and mechanisms, and its practical applications in ECL detection are sparse and limited in scope. Further research and exploration are needed to harness its potential in ECL-based applications fully.

#### 2.1.3. Nanomaterial-Based ECL Luminophores

In recent decades, with the development of nanomaterials, the ECL phenomena of many nanomaterials have been reported. Due to their excellent chemical, physical, and physicochemical properties, ECL nanomaterials have attracted widespread attention and found extensive applications. In 2002, the first ECL nanomaterial, semiconductor silicon nanocrystals, was reported [72]. Since then, a series of nanomaterials exhibiting ECL properties has been synthesized, becoming a hot research topic in the field of ECL. Currently, research on ECL nanomaterials is primarily focused on several areas, including quantum dots, metal nanoclusters, organic polymer nanoparticles, and others. Additionally, other types of nanomaterials, such as composites of luminescent molecules and nanomaterials, MOF nanomaterials, and metal oxide nanomaterials, are also being explored for use in ECL applications.

**Figure 3:**
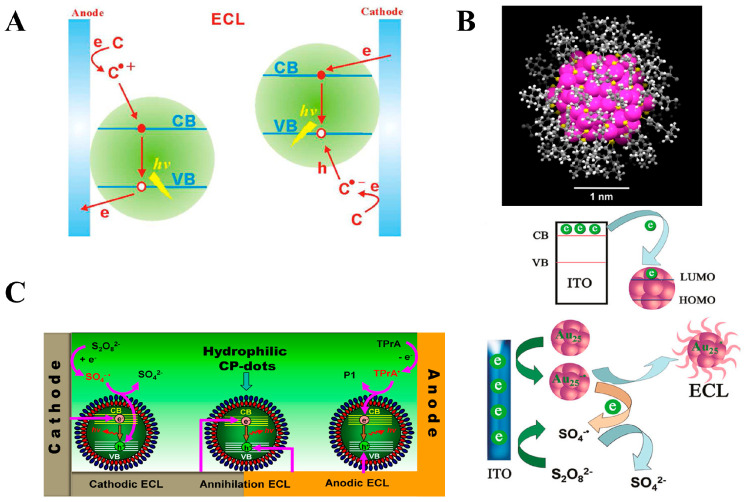
(**A**) ECL mechanism of the co-reactant pathway for quantum dots [75]; (**B**) structure of metal nanoclusters and their ECL mechanism via the co-reactant pathway [78]; (**C**) ECL mechanism of organic polymer nanoparticles [84,85]. * The formation of excited-state.

Quantum dots are zero-dimensional nanomaterials with unique optoelectronic properties. The size of quantum dots is generally smaller than or comparable to the Bohr radius of their excitons, where their distinct physical properties are governed by the quantum confinement effect, including surface effects, photoelectric effects, and quantum effects [73,74,75,76]. In recent years, due to their unique optoelectronic properties, quantum dots have been widely applied in the field of ECL research. Quantum dots with ECL properties have been sufficiently reported, and this research has been categorized into two main types: semiconductor-based quantum dots, such as Si, Ge, and CdSe, and carbon-based quantum dots, including carbon quantum dots and graphene oxide quantum dots. The ECL mechanisms of quantum dots involve two reaction pathways: the annihilation pathway and the co-reactant pathway. The basic process of the annihilation pathway is consistent with the processes described by Equations (1)–(5). The co-reactant pathway can be summarized as shown in Figure 3A [75]. At the anode, the electrode induces holes in the valence band (VB) of the quantum dot, while the co-reactant undergoes an oxidation reaction, injecting electrons into the system. The recombination of holes and electrons then results in anodic ECL. At the cathode, the electrode and co-reactant interact to facilitate electron–hole exchange, generating cathodic ECL. The discovery of the ECL properties of quantum dots has expanded ECL research into the field of nanomaterials, marking a milestone in the development of ECL studies. Subsequently, many researchers have focused on the application of ECL nanomaterials, exploring the use of quantum dots in practical applications such as bio-sample analysis, light-emitting diode systems, environmental protection, energy, and catalysis. Additionally, many new nanomaterials, including metal nanoclusters, have been synthesized for ECL research.

Metal nanoclusters consist of atomic clusters composed of a few to approximately 100 atoms, typically ranging in size from subnanometer (<2 nm) to several nanometers. Metal nanoclusters can be divided into two parts: the metal core and the organic ligands that act as protecting agents (Figure 3B) [77,78]. The size of the nanocluster is related to the number of atoms in the cluster. It is comparable to the electronic Fermi wavelength of the metal atoms, thereby exhibiting unique photoelectric properties, such as excellent optical, electrical, electrochemical, and chemical characteristics. Over the past decade, due to their superior optical, electrical, and chemical properties, metal nanoclusters have attracted significant attention from researchers in the field of ECL. A series of reports on the ECL properties of metal nanoclusters has emerged. The first reported metal nanoclusters with ECL properties were silver (Ag) nanoclusters, followed by the synthesis of gold (Au), copper (Cu), platinum (Pt), palladium (Pd), and alloy nanoclusters, with the associated ECL mechanisms gradually maturing. The ECL mechanisms of metal nanoclusters are classified into two pathways: the annihilation pathway and the co-reactant pathway. The annihilation pathway is similar to the mechanism of quantum dots. The co-reactant pathway is as follows: Au nanoclusters form an anionic free radical at the electrode surface, while the co-reactant undergoes oxidation at the electrode surface, generating free radical ions. These species then undergo electron transfer, forming excited-state Au nanoclusters, which return to the ground state and emit light (Figure 3B). Studies have shown that, compared to other nanoclusters, Au nanoclusters possess unique physicochemical properties, such as low toxicity, easy labeling, and good stability, making them highly popular in ECL analytical applications [79]. In contrast, the ECL performance of Ag, Cu, and other nanoclusters is inferior to that of Au nanoclusters, with only a few reports in the literature [80,81,82,83], and further research is needed. Additionally, alloy nanoclusters are attracting growing interest. The formation of alloy nanoclusters with multiple metals may yield ECL materials with superior properties. However, research in this area is still in its early stages, requiring substantial efforts from the research community.

Organic polymer nanoparticles refer to nanomaterials with particle sizes ranging from 1 to 100 nm, formed by the polymerization of organic monomers. The ECL phenomenon of organic polymer nanoparticles was first reported in 2003 [86], which subsequently attracted considerable attention from researchers. As research on organic polymer nanoparticles progressed, it was found that, compared to other ECL nanomaterials, organic polymer nanoparticles possess advantages such as ease of functionalization, good biocompatibility, and low toxicity [84], which has led to increasing interest from researchers in studying the ECL properties of these materials. The ECL mechanism of organic polymer nanoparticles follows the same principles as other luminophores, involving both the annihilation pathway and the co-reactant pathway, as described below (Figure 3C) [83,84]. In the annihilation pathway, ECL is generated by the transfer of electrons and holes within the organic polymer nanoparticles. In the co-reactant pathway, anodic ECL is induced when the electrode directly injects holes into the valence band of the organic polymer nanoparticles. At the same time, electrons in the conduction band are provided by the free radicals of the co-reactant. Cathodic ECL occurs when the electrode directly provides electrons, and the holes are supplied by the ${\mathrm{SO}}_{4}^{•-}$ radicals generated at the cathode. Up to now, researchers have employed various methods and organic monomers to synthesize organic polymer nanoparticles with desirable characteristics, such as good water solubility, tunable colors, and low potential [87,88,89], enriching the field of ECL research and opening new directions for further development.

In addition to the ECL nanomaterials mentioned above, other types of nanomaterials have also been reported to exhibit ECL properties. These materials include (1) luminescent molecule and nanomaterial composites, such as Ru-doped SiO_2_ nanoparticles and gold nanoparticles reduced by luminol [90,91], (2) MOF nanomaterials, such as MOF-5, Eu-MOF, ZnTCPP, etc. [92,93,94], (3) metal oxides, such as TiO_2_ nanoparticles, ZnO microspheres, and SnO_2_ nanocrystals [95,96,97], and (4) two-dimensional nanomaterials, such as g-C_3_N_4_ and graphene nanosheets.

### 2.2. Potential-Resolved ECL

Although numerous ECL luminophores have been developed, studied, and utilized, most of these studies are limited to single luminophores that emit only one ECL signal. In recent decades, three main types of potential-resolved ECL systems have been reported: metal–organic complexes, layer-by-layer-modified electrodes, and nanomaterials. However, the multiple ECL emissions from a single luminophore are quite limited. Therefore, the development of novel nanoluminophores that exhibit multiple ECL emissions is significant for advancing ECL technology and its applications in detection.

#### 2.2.1. Potential-Resolved ECL of Metal–Organic Complexes

**Figure 4:**
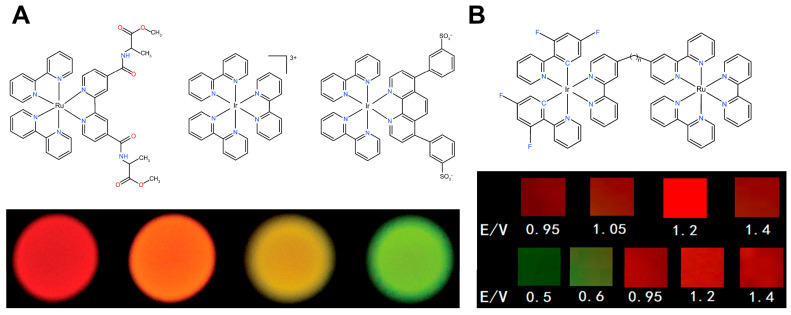
(**A**) ECL with potential-resolved multicolor emission of mixed organic complexes of Ru^2+^ and Ir^3+^ [99]; (**B**) ECL luminophore based on multinuclear metal complexes and their potential-resolved multicolor ECL [102].

The potential-resolved ECL system based on metal–organic complexes can be primarily categorized into two types. The first type is the ECL system of mixed metal–organic complexes, which represents a pioneering research direction in this field, and the mechanism relies on the potential-gated cooperative luminescence of heterogeneous luminophores. In 2004, Muegge and co-workers achieved the first potential-resolved ECL of a [Ru(bpy)_3_]^2+^/[Ir(ppy)_3_]^3+^ mixture in an acetonitrile solvent system. By precisely controlling the molar ratio of the two components, they successfully captured a potential-dependent ECL spectral image (Figure 4A) [98,99]. However, this type of system faces two major technical challenges: (1) most metal complexes (such as Os, Re series) suffer from poor water dispersibility and insufficient biocompatibility, which limits practical applications to only Ru^2+^/Ir^3+^ complexes, and (2) the inherent spectral emission singularity severely restricts their multicolor encoding capacity. These limitations significantly hinder the development of potential-resolved multicolor ECL in metal–organic complexes. A breakthrough in this area came from Park’s group, who proposed a ligand engineering strategy [99,100,101]. By introducing π-extended ligands (e.g., 4,4′-dicarboxy-2,2′-bipyridine), the ECL emission of Ru^3+^ complexes could be red-shifted by up to 65 nm. In contrast, the use of rigid planar ligands (e.g., benzimidazole derivatives) could enable blue-shift control of Ir^3+^ complexes. This ligand-dependent luminescence tuning mechanism provides a theoretical framework for the development of novel metal–organic luminophores with excellent water dispersibility and multicolor ECL response. It holds significant value for advancing ECL technology in vivo sensing and multi-target detection applications.

The second class of systems focuses on multinuclear metal–organic ECL luminophores, whose core design strategy involves the covalent bonding of two or more heterometallic centers (e.g., Ru^2+^/Ir^3+^, Ru^2+^/Os^2+^) via bridging ligands, thereby constructing multinuclear metal complex ECL luminophores centered around multiple metal ions (Figure 4B) [102]. Steve Welter et al. made a groundbreaking advancement in this field by successfully synthesizing a Ru^2+^-Ir^3+^ dinuclear complex using a bidentate bipyridyl carboxylate ligand, achieving the first potential-resolved dual-color ECL at the molecular level [103]. This system controls intramolecular charge transfer efficiency by regulating the intermetallic distance, resulting in a linear response of the intensity ratio between the dual emission wavelengths as a function of potential. However, the synthesis of such compounds is complex, and the selection of bridging ligands remains challenging, with only a few studies reporting ECL luminophores of this type. Despite these challenges, the unique advantages of multinuclear complexes—such as the programmability of excited intramolecular states (through metal combinations and ligand engineering to regulate emission band position and intensity ratio)—provide a theoretical framework for the development of novel potential-wavelength dual-dimensional-encoded ECL probes, which could have potential in super-resolution imaging and multiplexed biosensing.

#### 2.2.2. Potential-Resolved ECL Based on Layer-by-Layer-Modified Electrodes

Potential-resolved ECL based on layer-by-layer-modified electrodes refers to the multiple potential-resolved or wavelength-resolved ECL emissions in a single electrochemical process by assembling different ECL luminophores onto the surface of an electrode via layer-by-layer modification. This system is primarily designed for highly accurate self-calibration detection and simultaneous multi-target detection, utilizing ECL signals at different potentials or wavelengths to reduce system errors through ratio-based self-calibration, thereby enabling precise detection and the recognition of multiple target molecules for simultaneous detection.

**Figure 5:**
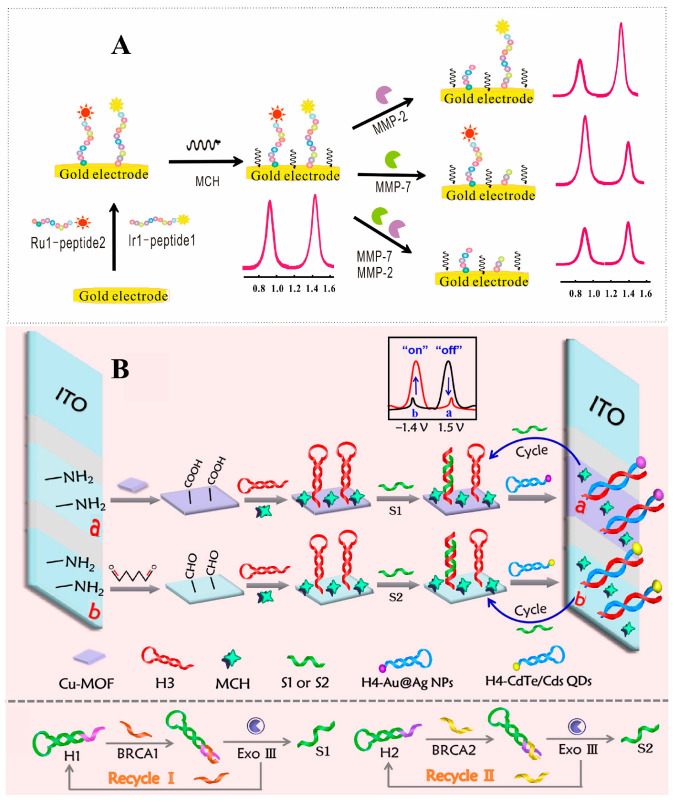
(**A**) Construction process of a potential-resolved layer-by-layer-modified electrode [104]; (**B**) Construction process of a spatial and potential-resolved layer-by-layer-modified electrode [105].

The first type of potential-resolved layer-by-layer-modified electrode involves modifying of two or more ECL luminophores with different wavelengths or potentials on a single electrode surface, which generates two or more distinguishable ECL signals in a single electrochemical process. Hongfang Gao and co-workers employed Ir^3+^ and Ru^2+^ organic complexes as potential-resolved ECL probes and designed a potential-resolved dual-marker biosensor for ECL-based detection [104]. They first designed and synthesized Ir^3+^ and Ru^2+^ organic complexes with carboxyl groups that emitted ECL at different potentials. These complexes were covalently linked to a peptide and subsequently modified onto a gold electrode, serving as a multicolor potential-resolved ECL signal source. The biosensor utilized protease-specific recognition of the peptide to cause the emission molecules to detach from the electrode surface, thereby reducing the ECL signal for detection. This biosensor (Figure 5A) enabled simultaneous detection of two matrix metalloproteinases (MMP-2 and MMP-7) with detection limits of 5 ng/mL and 10 pg/mL, respectively [104].

The second type of spatially and potential-resolved layer-by-layer-modified electrode involves first dividing the electrode surface into different reaction regions and then modifying each region with different wavelength or potential ECL luminophores, enabling the generation of two or more distinguishable ECL signals in a single electrochemical process. Qianqian Cai synthesized a novel self-luminescent copper-based metal–organic framework (Cu-MOF), which can generate excellent positive-potential ECL without the need for co-reactants (Figure 5B) [105]. To achieve both positive and negative potential ECL simultaneously, Cu-MOF and CdTe@CdS quantum dots were integrated at different locations on an indium tin oxide (ITO) electrode, generating potential-resolved ECL using only a single co-reactant. The Cu-MOF was placed in channel 1 as a signal luminophore, and Au@Ag NPs were used to quench the positive-potential ECL of Cu-MOF via resonance energy transfer (RET) for the detection of the target BRCA1. Similarly, CdTe@CdS quantum dots were introduced into channel 2 as a negative-potential ECL material for the detection of BRCA2. This work not only developed a novel ECL material but also proposed an ingenious strategy for multi-target simultaneous detection, which holds broad application prospects in the field of biochemical analysis.

#### 2.2.3. Potential-Resolved ECL Based on Nanomaterials

ECL nanomaterials have become one of the key research areas in the field of ECL, and the synthesis of potential-resolved ECL nanomaterials has emerged as an important and emerging hotspot in this field. Potential-resolved ECL nanomaterials refer to those that generate multiple ECL emissions with potential resolution and different wavelengths in a single electrochemical process. With the development of analytical detection technologies, monochromatic or single-emission ECL nanomaterials can no longer meet the demands of current analytical advancements. Therefore, the development of multi-emission ECL nanomaterials has attracted considerable attention from researchers.

**Figure 6:**
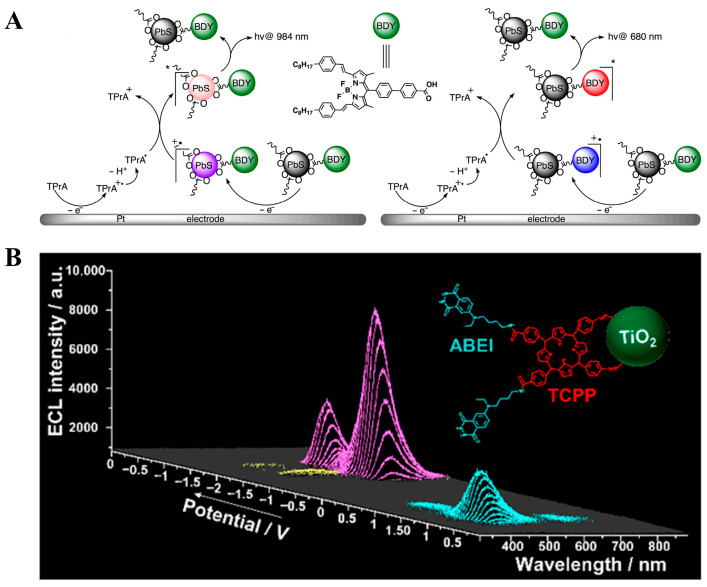
(**A**) ECL mechanism of PbS nanocrystals encapsulated with the fluorescent dye BODIPY [106]; (**B**) ECL properties of potential-resolved multicolor ECL nanomaterials with three different emission colors in aqueous solution [24]. * The formation of excited-state.

In 2015, Mahdi Hesari et al. [106] synthesized for the first time a potential-resolved ECL nanomaterial by encapsulating PbS nanocrystals with the fluorescent dye boron-dipyrromethene (BODIPY). ECL properties were investigated, and the corresponding ECL mechanisms were proposed. The results showed that this luminophore produced ECL peaks at 679 nm and 984 nm at different potentials in organic solvents, with the two ECL emissions originating from the PbS nanocrystals and the BODIPY dye, respectively (Figure 6A) [106]. However, this ECL system could only operate in organic solvents, and the potential resolution was not fully achieved, significantly limiting its practical analytical applications. Therefore, the synthesis of water-soluble potential-resolved ECL nanomaterials is of great significance for the practical application of potential-resolved multicolor ECL. Subsequently, Jiangnan Shu and co-workers successfully modified TiO_2_ nanoparticles with ABEI molecules and 5,10,15,20-tetra(4-carboxyphenyl) porphyrin (TCPP) molecules (Figure 6B) [24], for the first time, to obtain a potential-resolved multicolor ECL nanomaterial that produces three different color emission peaks in an aqueous solution. This nanomaterial produced ECL peaks at 458 nm, 686 nm, and 529 nm at different potentials during a cyclic voltammetry scan in a mixed solution containing potassium persulfate (K_2_S_2_O_8_) and hydrogen peroxide (H_2_O_2_). The studies of ECL performance indicated that the three ECL peaks originated from ABEI, TCPP, and TiO_2_, respectively. Subsequently, this research group developed a series of similar ECL nanomaterials, such as the synthesis of ABEI-functionalized g-C_3_N_4_ by Jue Wang et al. This nanomaterial, using H_2_O_2_ and K_2_S_2_O_8_ as co-reactants, produced two ECL emissions at the cathode and anode during a cyclic voltammetry scan [107]. Dexin Du and colleagues encapsulated CdS quantum dots into MOF-5 [26] and, using K_2_S_2_O_8_ as a co-reactant, generated potential-resolved dual ECL peaks at negative potentials.

Wei Zhang and co-workers synthesized potential-resolved multicolor ECL supramolecular nanomaterials through the hydrogen bonding self-assembly of 3,4,9,10-perylene tetracarboxylic dianhydride (PTCDA) and aniline (An) [108]. This supramolecular nanomaterial reacted with a co-reactant solution of potassium persulfate (K_2_S_2_O_8_) in water, producing three ECL peaks at 486, 692, and 760 nm, corresponding to two different colors. The ECL mechanism was studied, and the results showed that the 486 nm emission originated from PTCDA, while the 692 nm and 760 nm emissions were derived from aniline.

## 3. Potential-Resolved ECL Applications

Potential-resolved ECL technology has been widely applied in the development of ratiometric sensing strategies since its inception. Compared to traditional monochromatic ECL systems, potential-resolved ECL luminophores can enable the selective activation of multiple wavelength signals in a single electrochemical scan, which successfully overcomes the technical limitations of traditional single-emission systems in cases such as simultaneous multi-target detection and real-time tracking of dynamic biological processes. To date, numerous potential ECL-based detection strategies have been reported. Based on multiple ECL signals of potential-resolved ECL, these strategies can be categorized into two main types: self-calibration strategies for precise analysis and simultaneous detection strategies for multi-target.

### 3.1. Applications in Self-Calibration Detection

Potential-resolved ECL has primarily been used in the research of ratiometric ECL sensors since its discovery. Due to the ability of ratiometric strategies to effectively eliminate errors caused by environmental changes, such as variations in light path, background light interference, and light scattering, these strategies significantly improve the sensitivity and accuracy of detection [25]. To date, numerous ratiometric ECL sensors have been successfully developed based on potential-resolved ECL. Recently, a differential detection strategy was reported, which effectively eliminates cumulative errors in the detection process by using the difference in the intensities of two ECL signals.

**Figure 7:**
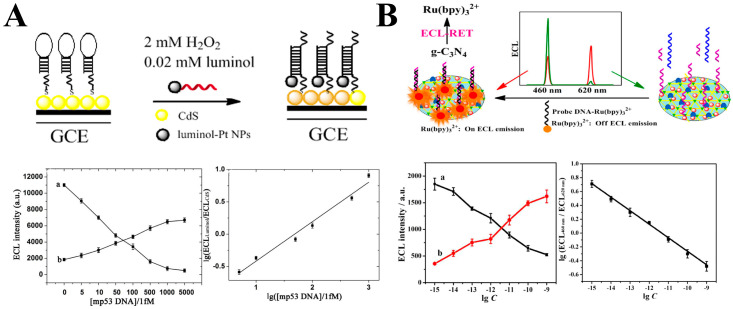
(**A**) Potential-resolved ratiometric ECL sensor: sensor construction process, ECL intensity variation with DNA concentration, and DNA detection calibration curve [109]; (**B**) Energy resonance transfer-based ratiometric ECL sensor: sensor construction process, ECL intensity variation with microRNA concentration, and microRNA detection calibration curve [110].

The first type of strategy is the dual-signal ratio method. This approach, inspired by ratiometric fluorescence analysis strategies, uses the ratio of two potential-resolved ECL signals (*I*_λ1_/*I*_λ2_) to design a ratiometric strategy, which can effectively eliminate errors caused by environmental changes (e.g., light path variations, background light interference, light scattering). The research group of Hongyuan Chen developed a dual-signal ratiometric ECL sensor using luminol-platinum nanoparticles and CdS quantum dots as potential-resolved multicolor ECL signal sources. By precisely tuning the energy level matching of luminol-platinum nanoparticles (anode emission, λ = 425 nm) and CdS quantum dots (cathode emission, λ = 530 nm), they achieved complete signal separation within a potential window, thereby constructing the first dual-signal ratiometric ECL sensor (Figure 7A). This system was successfully applied to ultrasensitive DNA detection, achieving a detection limit of 5.0 fM and a relative standard deviation (RSD) of 2.3% [109]. Subsequently, the research group developed a series of potential-resolved ratiometric ECL sensors for biological detection and analysis, laying the foundation for the ratiometric ECL strategy.

The second strategy focuses on the RET mechanism, utilizing the ratio of two different wavelength signals obtained through energy resonance transfer to establish a ratiometric strategy. Jingjuan Xu’s research group successfully assembled a wavelength-resolved ratio sensor using ECL luminophores g-C_3_N_4_ and Ru(bpy)_3_^2+^ as signal sources [110]. In this system, energy resonance transfer occurs, where g-C_3_N_4_ first generates ECL at the same excitation potential, and the energy is transferred to Ru(bpy)_3_^2+^, producing two wavelength-resolved ECL peaks. By utilizing the ratio of the two ECL signals, this strategy successfully achieves specific recognition of microRNA, improving sensitivity by 3.2-fold over the traditional molecular beacon method (Figure 7B) [110]. Subsequently, numerous ratiometric ECL sensors have been developed for detecting various target substances, including DNA, RNA, disease biomarkers, and heavy metal ions.

**Figure 8:**
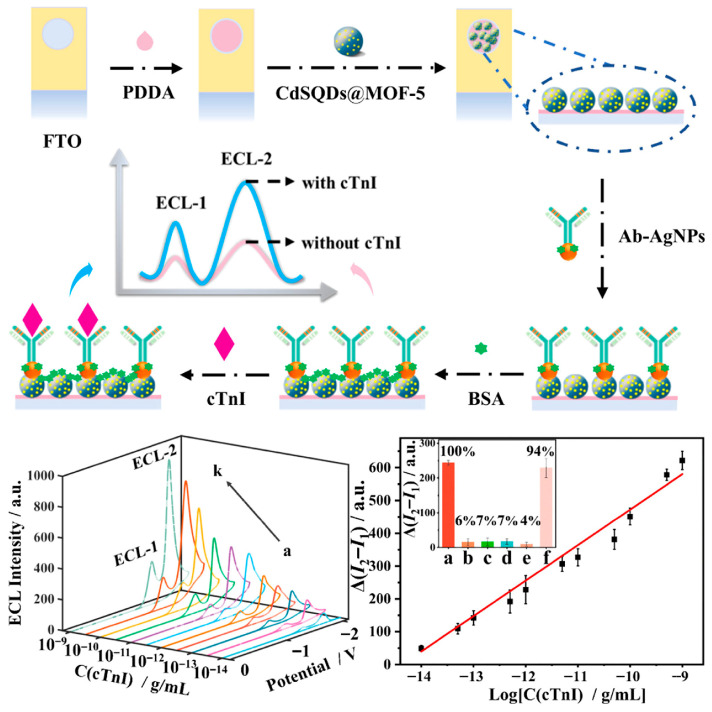
Construction process of the differential ECL immunosensor and its analytical performance [26].

The third strategy is the differential processing method, which is based on the difference between two potential-resolved ECL signals (Δ*I* = *I*_1_ − *I*_2_), establishing a differential detection strategy. By utilizing the signal difference, this method can eliminate cumulative errors caused by environmental changes (e.g., temperature fluctuations), matrix variations, pH fluctuations, and other factors. Dexin Du and co-workers constructed a CdS@MOF-5 ECL system by encapsulating CdS quantum dots within MOF-5 nanomaterials to obtain potential-resolved ECL luminophores. Under a single cyclic voltammetry scan, this system generated dual-color ECL emissions at −1.0 V (λ = 525 nm, CdS band-edge emission) and −1.4 V (λ = 650 nm, MOF defect-state emission). Based on this, a label-free differential ECL immunosensor was developed for the highly sensitive detection of cardiac troponin I in clinical serum samples (Figure 8) [26], providing a new direction for ECL sensor research and advancing the development of biomarker analysis and detection.

### 3.2. Applications in Simultaneous Multi-Target Detection

**Figure 9:**
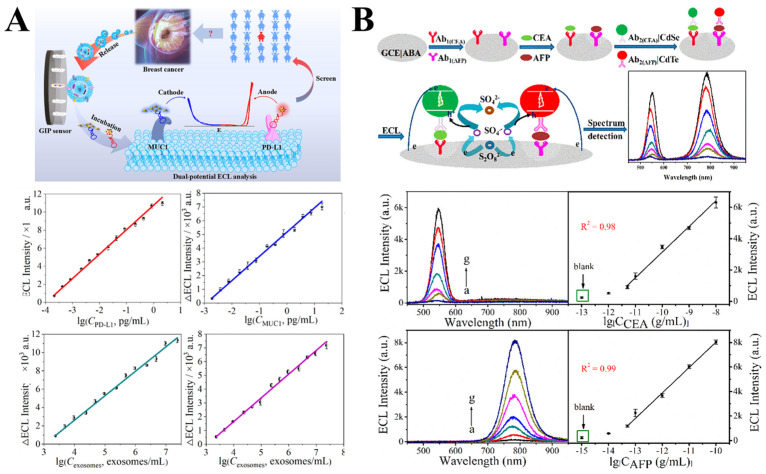
(**A**) Potential-resolved sandwich immunosensor for simultaneous detection of two biomarkers [112]; (**B**) wavelength-resolved sandwich immunosensor for simultaneous detection of two biomarkers [113]. Blank represents the control group without detection target.

Multi-target simultaneous detection offers numerous advantages in practical applications, including the conservation of reagents, a reduction in detection time, and the acquisition of more informative results. These benefits have attracted numerous researchers to improve the development of and research into multi-target detection technologies. In recent years, with the research and development of ECL luminophores, potential-resolved ECL strategies for simultaneous multi-target detection have garnered widespread attention. Potential-resolved ECL has many practical advantages in multi-target detection, including low cost, high sensitivity, and good controllability, making it an attractive approach for multi-target detection [111]. Currently, the most widely reported strategies for simultaneous multi-target detection are based on potential-resolved ECL with layer-by-layer-modified electrodes, which can be categorized into three main types:

The first type is the potential-resolved strategy, where ECL signals generated at different applied potentials are used for the recognition and simultaneous detection of multiple targets. Kui Luo and co-workers constructed a glycosylation imprinting ECL sensor (Figure 9A) to capture breast cancer exosomes by adsorbing the polysaccharides of exosome PD-L1 onto glycosylation-imprinted polymers (GIP) [112]. Then, Au@luminol-PD-L1 and Au@g-C aptamer probes were used to label PD-L1 and MUC13N4-MUC1 specifically. The GIP membrane was used to identify breast cancer exosomes, and the potential-resolved ECL signals from the probes were recorded at cathodic (−1.4 V) and anodic (+0.7 V) potentials, respectively. This platform enabled the quantification of exosomes in breast cancer and the detection of exosome biomarkers PD-L1 and MUC1. Although subsequent studies have reported biosensors based on potential-resolved ECL signals for detecting various targets, the simultaneous detection strategy of multiple targets remains a challenging task, and the related research is still limited.

The second type is the wavelength-resolved strategy, where different wavelength ECL emission signals are used to differentiate between various targets, which enables simultaneous multi-target detection. Guizheng Zou and co-workers used CdTe and CdSe nanocrystals (NCs) with different emission wavelengths as labels, which were attached to secondary antibodies as wavelength-resolved ECL signal probes, to construct a sandwich-type wavelength-resolved immunosensor for the simultaneous detection of two biomarkers (Figure 9B) [113]. At the maximum wavelengths of 776 nm and 550 nm, the sensor simultaneously detected carcinoembryonic antigen (CEA) and alpha-fetoprotein (AFP), with detection limits of 1 pg/mL and 10 fg/mL, respectively. This study successfully demonstrated the capability of the wavelength-resolved ECL strategy to simultaneously detect multiple biomarkers in a single test, exhibiting a higher sensitivity and signal-to-noise ratio compared to other analytical techniques. However, compared to the other potential-resolved strategies, this approach relies on expensive spectral analysis systems to interpret the different wavelength signals, which limits the further development and application of this strategy. Therefore, developing simple, cost-effective, and rapid detection strategies holds significant practical importance.

**Figure 10:**
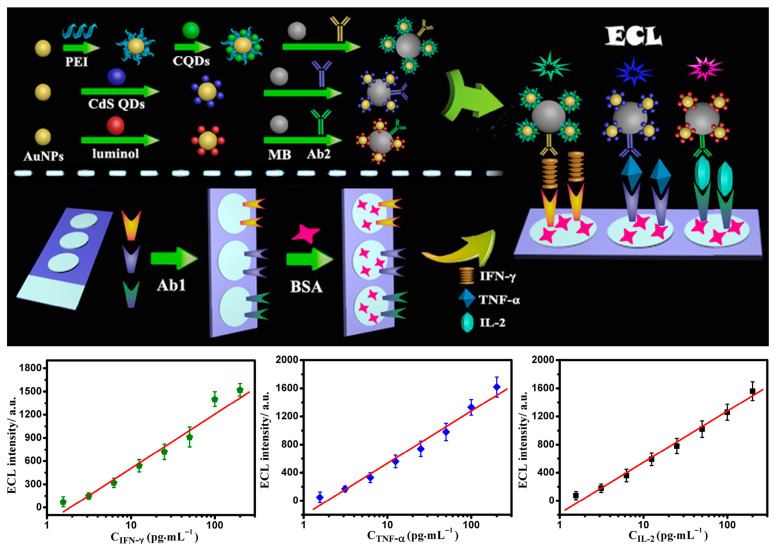
Strategy of the potential and spatial-resolved sandwich immunosensor for the simultaneous detection of three biomarkers, depicted as a calibration curve [114].

The third strategy is the spatial-resolved approach, which constructs independent signal recognition channels based on the spatial location differences in the sensing interface, enabling the simultaneous analysis of multiple targets. Bin Zhou et al. developed a sandwich-type ECL immunosensor capable of simultaneously detecting three biomarkers (Figure 10) [114]. The key to this sensor lies in the spatially encoded design of the sensing interface: by fixing three ECL probes with potential-resolved properties (such as luminol, carbon quantum dots (CQDs), and CdS quantum dots) at different spatial regions of the electrode, they constructed a multi-channel detection platform with both potential-resolved and spatial-resolved capabilities. In a single cyclic voltammetry scan, the sensor can simultaneously achieve highly sensitive detection of tuberculosis infection biomarkers, including interferon-γ, tumor necrosis factor-α, and interleukins, by exploiting differences in both potential (excitation potentials of different probes) and spatial positioning (regional arrangement of probes). The detection limits for all targets were as low as 1.6 pg/mL. Notably, the sensor demonstrated excellent performance in detecting clinical serum samples, which was highly consistent with the standard ELISA method. These results provided strong evidence for reliability in complex biological matrices.

## 4. Conclusions and Outlook

ECL detection technology, with its advantages of simple equipment, high sensitivity, and wide detection range, has been widely applied in the analysis of biological samples (such as immunoassays, nucleic acid, and small-molecule metabolite detection). However, ECL detection is prone to interference and struggles to achieve high-throughput detection, which limits its practical application. Therefore, developing precise ECL strategies and multi-target ECL strategies is crucial. To date, research in this field has focused on two main strategies: ECL of layer-by-layer-assembled modified electrodes and potential-resolved ECL of nano-luminophores. The former typically requires complex labeling and modification to integrate multiple luminophores onto the electrode surface, generating potential-resolved ECL. Although this approach can be used for precise analysis and multi-target detection, the construction process of layer-by-layer-assembled modified electrodes is cumbersome and time-consuming. In contrast, ECL luminophores in the latter strategy can inherently generate multi-potential/multi-wavelength ECL emissions without the need for complex assembly, thereby achieving precise analysis and multi-target detection strategies with the advantages of simplicity, time-saving, and efficiency. However, research in this field is still in its early stages, and the variety of available materials is limited. There is an urgent need to design novel potential-resolved ECL luminophores and develop new strategies based on these materials that integrate precise analysis, high-throughput capabilities, and multi-target detection to advance the practical applications of ECL and label-free detection technologies.

To address this challenge, future research should focus on two interconnected fronts: novel luminophore design and innovative detection strategies. For the former, a rational design principle involves precisely tuning the electrochemical and spectral properties of materials. This can be achieved through several approaches: First, molecular engineering via systematic modification with electron-donating/withdrawing groups or heteroatom doping can fine-tune HOMO/LUMO energy levels and band gaps to create novel potential-resolved ECL luminophores with distinct redox potentials. Second, the development of multi-emissive systems (e.g., composited, modified, or doped nanomaterials) that incorporate spatially separated emitting centers can generate resolved ECL signals. Finally, the exploration of emerging materials, such as covalent organic frameworks (COFs) and metal–organic frameworks (MOFs), offers unparalleled opportunities for designing resolved ECL luminophores due to their periodic and tunable structures for integrating multiple, well-defined ECL centers. Concurrently, new detection protocols must be developed. The promising approaches design spatial and temporal encoding strategy by integrating them with microfluidic arrays, intelligent signal processing coupled machine learning to achieve high-throughput multi-detection and precision diagnostics. The final goal is to create intelligent, label-free ECL platforms that seamlessly integrate precise analysis, high-throughput capability, and multi-target detection.

In summary, potential-resolved ECL technology holds transformative prospects in the medical field. The key advantage lies in the ability to perform self-calibration detection and simultaneous multi-target detection, which promotes the development of precision diagnostics toward greater efficiency and dynamic capabilities. In the early diagnosis of diseases, detection platforms based on potential-resolved ECL luminophores can simultaneously capture a range of low-abundance biomarkers (such as circulating tumor DNA, exosome proteins, etc.), combined with artificial intelligence-assisted potential-resolved signal analysis, where the early screening and subtype diagnosis of complex diseases such as cancer and neurodegenerative diseases will be promising. In the field of personalized treatment, this technology can be integrated into microfluidic chips or wearable devices to monitor the dynamic changes in drug-metabolizing molecules or inflammatory factors in the blood or interstitial fluid of patients in real-time, providing immediate data support for precision dosing and efficacy assessment. Additionally, with breakthroughs in novel ECL luminophores and flexible bioelectronics, ECL detection technology will be further integrated with portable medical devices, promoting the widespread use of bedside detection and home health monitoring. It could even be used to create an intelligent disease warning network through multi-modal signal fusion technologies (such as ECL–electrochemical–optical coupling), providing key technical support for the digital transformation of healthcare.

## Data Availability

No new data were created or analyzed in this study.

## Abbreviations

The following abbreviations are used in this manuscript:

ECL	Electrochemiluminescence
ABEI	*N*-(4-aminobutyl)-*N*-ethylisoluminol
MOF	metal–organic framework
VB	valence band
MMP	matrix metalloproteinases
Cu-MOF	copper-based metal–organic framework
ITO	indium tin oxide
RET	resonance energy transfer
BODIPY	boron-dipyrromethene
TCPP	5,10,15,20-tetra(4-carboxyphenyl) porphyrin
PTCDA	3,4,9,10-perylene tetracarboxylic dianhydride
RSD	relative standard deviation
GIP	glycosylation-imprinted polymers
NCs	nanocrystals
CEA	carcinoembryonic antigen
AFP	alpha-fetoprotein
